# Quantifying the Performance of Individual Players in a Team Activity

**DOI:** 10.1371/journal.pone.0010937

**Published:** 2010-06-16

**Authors:** Jordi Duch, Joshua S. Waitzman, Luís A. Nunes Amaral

**Affiliations:** 1 Department of Chemical and Biological Engineering, Northwestern University, Evanston, Illinois, United States of America; 2 Northwestern Institute on Complex Systems, Northwestern University, Evanston, Illinois, United States of America; 3 Department of Computer Science and Mathematics, Universitat Rovira i Virgili, Tarragona, Spain; 4 Howard Hughes Medical Institute, Northwestern University, Evanston, Illinois, United States of America; University of East Piedmont, Italy

## Abstract

**Background:**

Teamwork is a fundamental aspect of many human activities, from business to art and from sports to science. Recent research suggest that team work is of crucial importance to cutting-edge scientific research, but little is known about how teamwork leads to greater creativity. Indeed, for many team activities, it is not even clear how to assign credit to individual team members. Remarkably, at least in the context of sports, there is usually a broad consensus on who are the top performers and on what qualifies as an outstanding performance.

**Methodology/Principal Findings:**

In order to determine how individual features can be quantified, and as a test bed for other team-based human activities, we analyze the performance of players in the European Cup 2008 soccer tournament. We develop a network approach that provides a powerful quantification of the contributions of individual players and of overall team performance.

**Conclusions/Significance:**

We hypothesize that generalizations of our approach could be useful in other contexts where quantification of the contributions of individual team members is important.

## Introduction

The importance of teams is nowadays widely accepted [1], [2]; we know that the composition of teams determines their odds of success [3], [4]. However, it is unclear how team processes lead to greater performance or how individual roles and strengths are combined for optimal results. Indeed, while the contributions of “superstars” are widely acknowledged [5], [6], their impact on the performance of their teams is far from having being established quantitatively. This raises the question: are the large disparities in compensation truly representative of the value that each individual brings to the team?

The main obstacle to answering this question has been our current inability to closely monitor individual actions of team members working together on different events. Team sports offer an extraordinary opportunity to overcome these challenges because interactions between team members are on display for a large number of events.

Soccer is widely viewed as the most popular sport world-wide. Soccer is also one of the most difficult sports to analyze quantitatively due to the complexity of the play and to the nearly uninterrupted flow of the ball during the match. Indeed, unlike baseball or basketball, for which there is a wealth of statistical performance data detailing how each player contributes to the final result, in soccer it is not trivial to define quantitative measures of an individual's contribution. Moreover, because soccer scores tend to be low, simple statistics such as number of assists, number of shots or number of goals only rarely provide a reliable measure of a player's true impact on the match's outcome. Instead, the real measure of the performance of a player is “hidden” in the plays of a team: a player can have tremendous impact by winning the ball from the other team or by passing to a teammate who then makes an assist.

Similarly to many other team activities, this type of information required to quantify in detail the role of a team member on team performance is not usually gathered and analyzed in a systematic way (for exceptions see [7], [8]). In the case of soccer, while the assignment of the credit is usually purely based on the subjective views of commentators and spectators, there typically exists a strong consensus on the quality of team play or of individual performances.

## Methods

The Euro Cup tournament is second only to the World Cup in terms of general interest, attracting millions of spectators and widespread media coverage. The 2008 tournament was unusual in the amount of statistical information that was collected and published online (see http://euro2008.uefa.com). This wealth of information enabled us to develop a new approach to quantify the performance of players and teams inspired by methods from social network analysis [9], [10].

To capture the influence of a given player on a match, we construct a directed network of “ball flow” among the players of a team. In this network, nodes represent players and arcs are weighted according to the number of passes successfully completed between two players. We also incorporate shooting information by including two non-player nodes, “shots to goal” and “shots wide”. A player's node is connected to these two nodes by arcs weighted according to the number of shots. We refer to the resulting networks as “flow networks”, and we build networks for the two teams in every match of the tournament.

**Figure 1:**
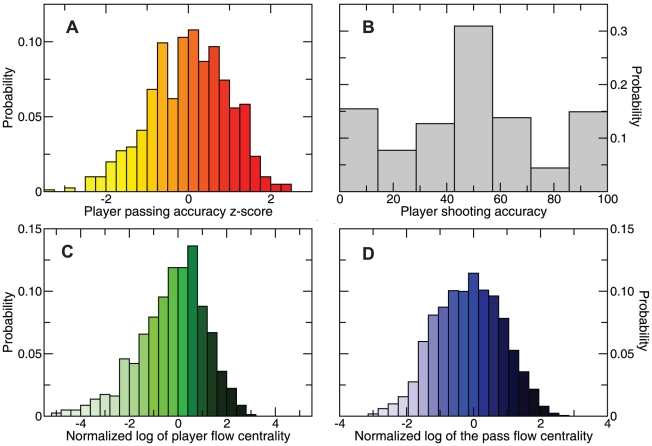
Performance statistics for individual players. (A) Distribution of the normalized player passing accuracy. We normalize the passing accuracy of each player that passed the ball at least 5 times during the match by the mean and standard deviation for the player's position. The mean (standard deviation) passing accuracy is 60.8 (15.7) for goalkeepers, 78.1 (10.1) for defenders , 75.6 (10.6) for midfielders, and 64.9 (12.8) for forwards. (B) Distribution of player shooting accuracy. We include only those players that shot the ball at least twice in a match. (C) Distribution of player performances. We define player performance as the normalized logarithm of the flow centrality (see text). We only include those players that passed the ball at least 5 times in a match. (D) Distribution of the normalized logarithm of the flow centrality for the passes (arcs) between players.

In order to obtain performance information, we start with the observation that a soccer team moves the ball with the opponent's goal in mind, keeping possession and shooting when the opportunity arises. A player's passing accuracy, which represents the fraction of passes initiated by a player that reach a teammate, and his shooting accuracy, which accounts for the fraction of shots that do not miss the goal, describe the capability of a player to move the ball towards the opponent's goal (Figs. 1A and 1B).

Combining the flow network with the passing and shooting accuracy of the players, we obtain the probability that each path definable on the network finishes with a shot. This procedure suggests a natural measure of performance of a player — the betweenness centrality [11] of the player with regard to the opponent's goal, which we denote as *flow centrality*. The flow centrality captures the fraction of times that a player intervenes in those paths that result in a shot. We take into account defensive efficiency by letting each player start a number of paths proportional to the number of balls that he recovers during the match. We define the *match performance* 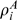 of player 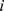 in team A as the normalized value of the logarithm of the player's flow centrality in the match (Figs. 1C and 1D).

## Results

### Team performance

**Figure 2:**
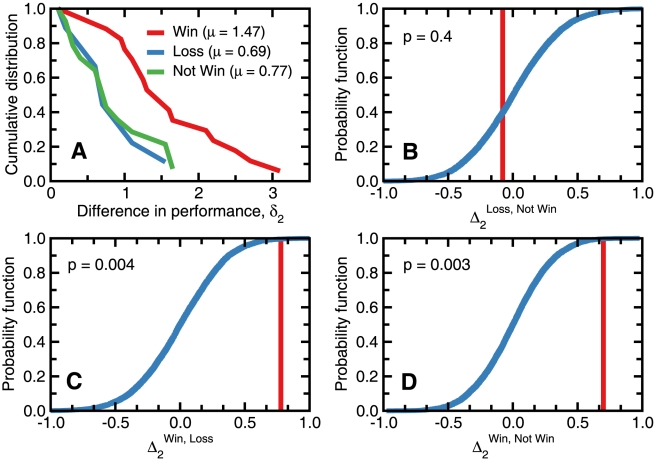
Validity of the flow centrality metric. We define team performance 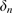 as the mean normalized log flow centrality of the 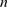 top players in a team. (A) Cumulative distribution of 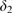 for matches where the team with highest performance wins, loses, or “not wins”. Clearly, the mean 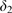 is much larger for games in which team with the highest performance wins. We use Monte Carlo methods with boostrapping to determine the significance of the differences in means for the different match outcomes. The red lines indicate the observed difference in 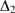 whereas the blue curves are the distribution of measured differences for the null hypothesis. (B) We find that there is no statistically significant difference in 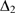 when comparing “Loss” versus “Not Win” outcomes. In contrast, we find highly significant differences when comparing (C) “Win” versus “Loss” or (D) “Win” versus “Not Win”.

**Figure 3:**
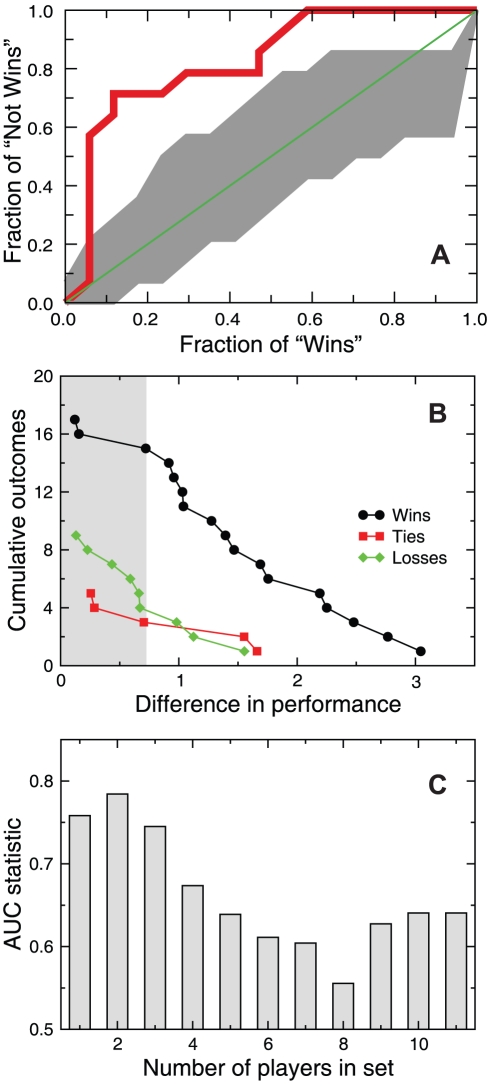
Sensitivity and specificity of the flow centrality metric. (A) For every distinct value of 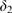 in our data, we calculate the fraction of values of 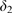 in the groups “Win” and “Not Win”. The area under the curve (AUC) statistic provides a measure of the sensitivity-specificity of the quantity under consideration [12]. Values of AUC close to 1 indicate high sensitivity with high specificity. We find an AUC of 0.825, much larger than the values expect by chance at the 90% confidence interval (shown in gray), which vary between 0.319 and 0.652. (B) Number of matches where the team with highest performance wins, ties, or loses as a function of 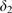. For the 20 matches where the difference is greater than 0.75, the team with the highest performance won 15 times, tied 2 and lost 3. This means that for 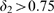 the odds of the team of highest performance winning the match are 3∶1. (C) AUC statistic as a function of 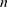 in 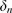 for “win” versus “Loss” outcomes. The highest AUC value is achieved for 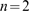.

We surmise that the player performance can be extended to the team level by calculating the average performance of a subset of players
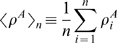
(1)where 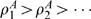. We further assume that performance differences between teams, which we define as
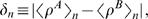
(2)will provide an indicator of which team “deserved” victory in a match (Fig. 2A). In order to test these hypotheses, we first obtain the distribution of differences in performance conditional on outcome
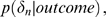
(3)where 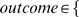
“Win”, “Loss”, “Not Win”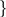. Figure 2 shows the cumulative distributions of 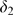 for these three outcomes (see Fig. 3 for a justification for this choice). It is visually apparent that there is a substantially larger mean 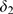 for the cases where the team with the highest performance wins the match.

We define 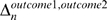 as
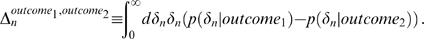
(4)To test the significance of the values of 
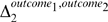
 obtained, we use bootstrap hypothesis testing [12]. Specifically, we pool the values of 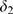 from all 30 matches in the tournament. We then draw surrogate random samples with replacement from the pooled data. For instance, for the case in Fig. 2B we draw surrogate “Loss” and “Not Win” samples with 9 and 14 data points, respectively, and then determine the difference in means of the two surrogate samples. We repeat this procedure 50,000 times in order to determine the significance of the observed 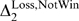. As shown in Figs. 2B, C, and D, we find that there is no significant difference in mean 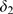 between “Loss” and “Not Win” outcomes, while the values of 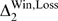 and 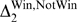 are highly significant (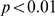).

The fact that 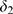 is significantly different for matches in which the team that wins has a better performance, suggests that the value of 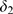 is correlated with the outcome of a match and thus can be used as an *objective* measure of performance. We thus use the area under the curve (AUC)—sometimes also called the receiver-operator curve (ROC) or the sensitivity-specificity curve—statistic in order to quantify the sensitivity and specificity of 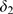. Figure 3A shows the AUC for the outcomes “Win” versus “Not Win.” We obtain an AUC of 0.825, which is far outside the 90% confidence band for random samples [0.319, 0.653]. We find that the best AUC value is found when team performance is defined as the average performance of the top two players in a team, although an average of the top 1 to 4 players would also lead to significant discrimination (Fig. 3B).

**Figure 4:**
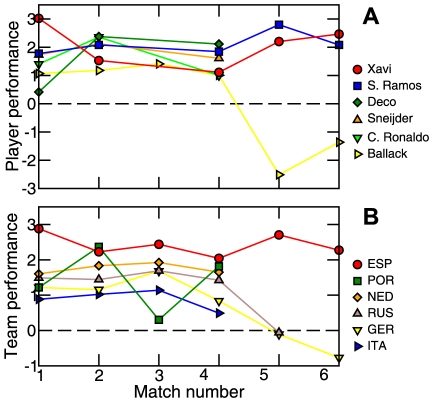
Time evolution of the performance of players and teams. (A) Xavi Hernandez, the MVP of the tournament, played extraordinarily well in the first match and in the tournament's final. The performance of Michael Ballack, the German team captain, is closely aligned with the performance of his team; as his performance slips in the knockout phase (games 4 to 6), Germany's performance also deteriorates. (B) Most teams performed at nearly constant levels during the first three matches of the tournament. In fact, the performance of a team during the first three matches was, for Euro 2008, a good predictor of the likelihood of a team winning the tournament.

The AUC analysis enables us to conclude that when 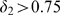, the odds that the team with higher performance wins the match are 3∶1 (Fig. 3C). Our team performance metric supports the general consensus that Spain, the winner of Euro 2008, played extremely well during the entire tournament (Table 1 and Fig. 4).

**Table 1:** Best team performances.

	Match performances			Tournament performances		Opponent performances	
Rank	Value	Team	Match	Value	Team	Value	Team
1	2.8	ESP	7	2.4	ESP	0.2	POR
2	2.7	ESP	30	1.7	NED	0.3	ESP
3	2.4	ESP	23	1.4	POR	0.3	SUI
4	2.3	POR	9	1.2	FRA	0.5	FRA
5	2.2	CRO	19	1.1	RUS	0.6	TUR
6	2.2	ESP	31	1.0	SUI	0.6	CRO
7	2.2	FRA	14	1.0	POL	0.7	AUT
8	2.2	ESP	15	0.8	ITA	1.1	ROU
9	2.0	ESP	28	0.8	CRO	1.2	NED
10	1.9	NED	21	0.6	GER	1.3	GER
11	1.8	NED	14	0.6	GRE	1.3	GRE
12	1.8	POR	25	0.5	AUT	1.3	POL
13	1.8	TUR	18	0.4	CZE	1.4	ITA
14	1.6	RUS	24	0.3	TUR	1.5	SWE
15	1.6	GER	20	0.2	SWE	1.6	CZE
16	1.6	NED	27	0.1	ROU	1.7	RUS

The ranking is in agreement with expert evaluations of the performance of the different teams. Note that all six matches played by Spain are in the top ten. The average performance of the opponents of a team provides a measure of defensive effectiveness. Note that Spain was able not only to perform very well but also to force its opponents to perform poorly, whereas Russia, for example, performed well but was unable to limit the play of its opponents.

### Individual performance

We next rank the performance of all the players of the tournament, and identify players who had influential contributions in a specific match or during the entire tournament. This comparison enables us to answer in an objective manner whether, for example, the most famous players fulfilled the expectations placed on them. We find that our metric provides sensible results that are in agreement with the subjective views of analysts and spectators (Table 2), demonstrating that our quantitative measure of performance captures the consensus opinions.

**Table 2:** Best individual performances.

	Match performances			Tournament performances	
Rank	Value	Player	Match	Value	Player
1	3.0	Xavi (ESP) *	7	2.1	S. Ramos (ESP)
2	2.7	S. Ramos (ESP)	30	2.1	Xavi (ESP) *
3	2.7	Villa (ESP) *	7	2.0	Senna (ESP) *
4	2.6	Silva (ESP)	30	1.9	Silva (ESP)
5	2.5	Alonso (ESP)	23	1.8	Sneijder (NED) *
6	2.5	Ribery (FRA)	14	1.6	Deco (POR)
7	2.5	Silva (ESP)	7	1.6	Capdevila (ESP)
8	2.4	Xavi (ESP) *	31	1.5	Ronaldo (POR)
9	2.3	Pranjic (CRO)	19	1.3	Villa (ESP) *
10	2.3	Deco (POR)	9	1.2	Petit (POR)
11	2.3	Senna (ESP) *	15	1.2	Fabregas (ESP) *
12	2.3	C. Ronaldo (POR)	9	1.2	Marchena (ESP) *
13	2.3	Fabregas (ESP) *	30	1.2	Inler (SUI)
14	2.3	De la Red (ESP)	23	1.1	Bosingwa (POR) *
15	2.2	Senna (ESP) *	7	1.1	Van der Vaart (NED)
16	2.2	Fabregas (ESP) *	23	1.0	Van Nistelrooy (NED)
17	2.2	Petit (POR)	9	1.0	Rakitic (CRO)
18	2.2	Xavi (ESP) *	30	1.0	De Jong (NED)
19	2.1	Rakitic (CRO)	19	0.9	Pavlyuchenko (RUS) *
20	2.1	Senna (ESP) *	28	0.9	Ooijer (NED)

Xavi Hernandez, who was named the tournament's MVP because of his performance in Spain's first match and the tournament final, and Sergio Ramos were lauded broadly for their performances. Spanish, Dutch and Portuguese players dominate both lists, in agreement with the consensus of many soccer analysts who identified these teams as the ones that played the best soccer. Indeed, a large number of the players on the list (marked with *) also appear in the “Team of the tournament” selected by the UEFA Technical Team.

Eight of the twenty players in our list of best performing players (Table 2) were also selected for the twenty-player team of the tournament. Note that we are excluding goal keepers from this analysis. Since the probability of a player being selected for the tournament team is 1/16 as there were 16 teams in the tournament, the probability of observing a given number of players from the tournament team in our top twenty is given by a binomial with 20 attempts and probability of 1/16. The probability of 4 or more players appearing in both lists by chance is approximately 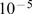. For all practical purposes, the probability of eight players appearing in both lists is zero.

### Performance visualization

**Figure 5:**
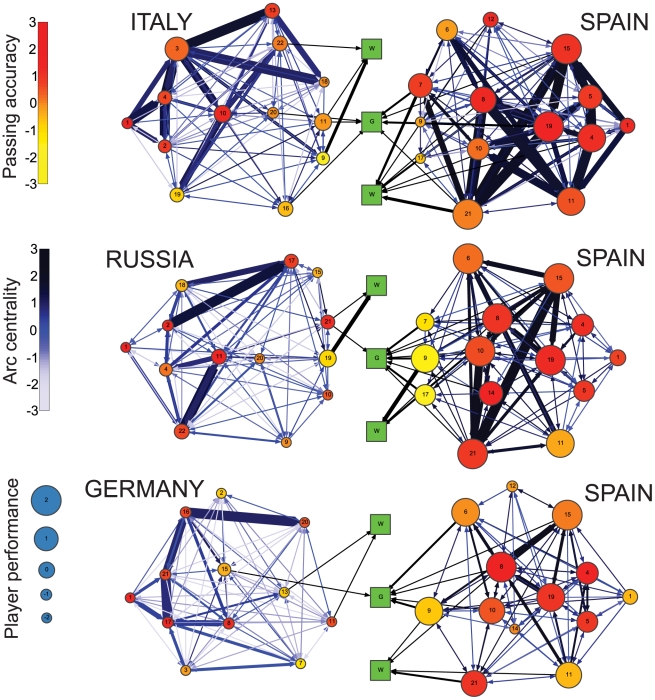
Visualization of the three knockout-phase matches of the Spanish team. Node position is determined by the player's field position and node number refers to the player's jersey number. Nodes are color-coded by the z-score of the passing accuracy of the player, and sized according to the player's performance. The width of the arcs grows exponentially with the number of passes successfully completed between two players, whereas the color indicates the normalized arc flow centrality. This representation of the “flow networks” allows us to encode a large amount of individual and team performance features enabling an observer to learn many aspects of a team's play.

The success of our performance metric in capturing the quality of play prompts us to develop a graphic representation of the play in a soccer match [13], [14]. We combine the network structure and the information compiled in the different distributions to display several features of a match that summarize the play during the 90 minutes (Fig. 5).

These representations enable us to compare the performance of the two teams in a given match and to identify the players with the most important roles during the match. Moreover, as the individual players' positions remain constant across networks, the different match networks can be easily compared to extract the general features of the play of a team, such as the efficiency of a particular team strategy.

### Extensions of our approach

Even though we developed and validated this approach for the case of soccer, we believe that it can be generalized to any team sport (or activity) where the final outcome is the result of a complex pattern of interactions among participants. In particular, the flow centrality metric we introduce may provide a new approach to quantify the contribution of individuals to teams working in other contexts. By combining information about skills, knowledge, and capabilities of the individuals, with information about the strength of the interactions between them —for example, using the number and length of phone calls or the number of e-mails exchanged— and information about completion of specific tasks, one could, potentially, quantitatively assess the individual performance of the team members and their contribution to the team's output.

In order to illustrate how our methodology could be extended to other activities that involve team work, we studied the interactions occurring in the process of completing several scientific projects that resulted in publications involving members of our lab. Specifically, we used email records to reconstruct the exchanges between the co-authors of the papers considered.

**Figure 6:**
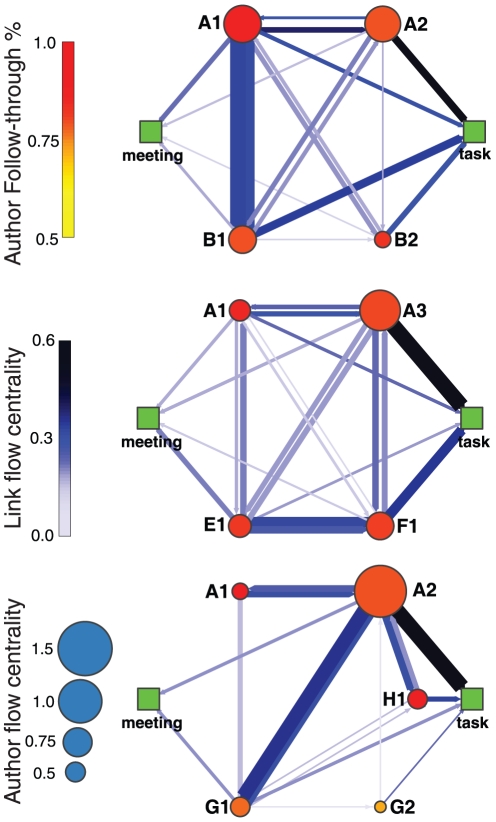
Visualization interactions between co-authors for three manuscript published by our lab. The letter in a node's label serves to distinguish labs, whereas the number serves to distinguish researchers within a lab. A node's label remains constant across projects and position is chosen for clarity of the representation. Nodes are color-coded by the z-score of the follow-through of the co-author, and sized according to the individual's flow centrality. The width of the arcs is proportional to the number of communications directed from one co-author to another, whereas the color indicates the arc flow centrality.

We then broke down these exchanges into path on the network of co-authors that terminate with (1) the completion of a task required for the paper, such as performing a calculation, obtaining some data, or writing some portion of the manuscript, (2) the scheduling of a meeting, or (3) the discarding of the task. This procedure enables us to build flow networks for each of the projects considered (Fig. 6). In these networks, a node represents a co-author in the manuscript, and the arcs represent the weighted communication directed from one co-authors to the other.

Additionally, we assign values to each of the completed task and scheduled meetings and award the corresponding value to each of the co-authors involved in the path. In this way, we are able to determine the flow centrality of each co-author in the project. Our analysis clearly reveals the different inputs and partitioning of responsibilities among co-authors for the different projects.

## Discussion

Our work demonstrates the power of social network analysis methods in providing insight into complex social phenomena. Indeed, whereas there are contexts in which simple measures or statistics may provide a very complete picture of an individual's performance —think of golf, baseball, or a track event— for most situations of interest, objectively quantifying individual performances or individual contributions to team performance is far from trivial.

At least in the context of a soccer, where quantification has always been challenging, we are able to demonstrate that flow centrality provides a powerful objective quantification of individual and team performance. While we cannot demonstrate the power of a similar approach in the context of a scientific collaboration, our preliminary results suggest that flow centrality does provide some insight into the variability in the partitioning of responsibilities among co-authors in a project.
